# Supplementary material for: Use of Whole-Genome Sequencing to Predict Mycobacterium tuberculosis Complex Drug Resistance from Early Positive Liquid Cultures
Source: Microbiol Spectr. 2022 Mar 21;10(2):e02516-21. doi: 10.1128/spectrum.02516-21 (PMC9045259; doi:10.1128/spectrum.02516-21)
Supplement: SUPPLEMENTAL FILE 1 — Supplemental material. Download SPECTRUM02516-21_Supp_1_seq6.pdf, PDF file, 0.1 MB [file spectrum02516-21_supp_1_seq6.pdf]

Supplementary Table 1 The amino acid mutations and frequency associated with drug resistance

Drug	Amino acid mutation	No. of isolates (%)
Rifampicin	*rpoB* Ser450Leu	35 (45.5)
	rpoB His445Arg	4 (5.2)
	rpoB His445Asn	3 (3.9)
	rpoB His445Asp	3 (3.9)
	rpoB Asp435Gly	2 (2.6)
	rpoB Asp435Tyr	2 (2.6)
	rpoB His445Tyr	2 (2.6)
	rpoB Leu430Pro	2 (2.6)
	rpoB Leu452Pro	2 (2.6)
	rpoB Leu464Met	2 (2.6)
	rpoB Ser450Phe	2 (2.6)
	rpoB Arg552Cys, *rpoB* Ser450Leu	1 (1.3)
	rpoB Asp435Gly, *rpoB* Leu452Pro, *rpoB* Leu464Met	1 (1.3)
	rpoB Asp435Tyr, *rpoB* His445Asp	1 (1.3)
	rpoB Asp435Val	1 (1.3)
	rpoB Gln432Lys	1 (1.3)
	rpoB Glu460Gly, *rpoB* His445Tyr, *rpoB* Ser450Leu	1 (1.3)
	rpoB His445Cys	1 (1.3)
	rpoB His445Tyr, *rpoB* Leu452Pro	1 (1.3)
	rpoB His445Tyr, *rpoB* Leu464Met	1 (1.3)
	rpoB His445Tyr, *rpoB* Ser450Leu	1 (1.3)
	rpoB Ile480Val, *rpoB* Ser450Leu	1 (1.3)
	rpoB Leu430Pro, *rpoB* Met434Ile	1 (1.3)
	rpoB Leu464Met, *rpoB* Ser450Leu	1 (1.3)
	rpoB Leu464Met, *rpoB* Ser450Tyr	1 (1.3)
	rpoB Ser441Leu	1 (1.3)
	rpoB Ser450Leu, *rpoC* Gly332Arg	1 (1.3)
	rpoB Ser450Leu, *rpoC* Ile491Thr	1 (1.3)
	rpoB Ser450Trp	1 (1.3)
Isoniazid	*katG* Ser315Thr	57 (75.0)
	fabG1 -15C>T	7 (9.2)
	ahpC -48G>A	1 (1.3)
	ahpC -48G>A, *katG* Ser315Thr	1 (1.3)
	ahpC -52C>T	1 (1.3)
	ahpC -54C>T, *katG* 2023_2024del	1 (1.3)
	ahpC -54C>T, *katG* Ser315Gly	1 (1.3)
	fabG1 -15C>T, *inhA* Ile21Thr	1 (1.3)
	fabG1 -15C>T, *katG* Ser315Thr	1 (1.3)
	fabG1 -8T>A, *katG* Ser315Thr	1 (1.3)
	fabG1 -8T>G, *katG* Ser315Asn	1 (1.3)

	katG Gly593Asp	1 (1.3)
	katG Ser160Leu, *katG* Ser315Thr	1 (1.3)
	katG Ser315Asn	1 (1.3)
Ethambutol	*embB* Met306Val	12 (23.1)
	embB Met306Ile	10 (19.2)
	embB Gln497Arg	6 (11.5)
	embA -12C>T	4 (7.7)
	embB Gly406Asp	4 (7.7)
	embB Asp354Ala	2 (3.8)
	embB His1002Arg	2 (3.8)
	embB Met306Leu	2 (3.8)
	embA -11C>A	1 (1.9)
	embA -11C>A, *embB* Met306Val	1 (1.9)
	embA -12C>T, *embB* Gly406Ala	1 (1.9)
	embA -12C>T, *embB* Tyr319Cys	1 (1.9)
	embA -16C>T, *embB* Gln497Arg	1 (1.9)
	embB Ala388Gly, *embB* His312Arg, *embB* Leu359Ile	1 (1.9)
	embB Asp1024Asn, *embB* Met306Ile	1 (1.9)
	embB Asp328Tyr	1 (1.9)
	embB Gln497Lys	1 (1.9)
	embB Gly406Cys	1 (1.9)
Streptomycin	*rrs* 799c>t, *rrs* 888g>a	24 (23.1)
	rpsL Lys43Arg	22 (21.2)
	rpsL Lys43Arg, *rrs* 799c>t, *rrs* 888g>a	10 (9.6)
	rrs 799c>t	8 (7.7)
	rrs 888g>a	7 (6.7)
	rpsL Lys43Arg, *rrs* 799c>t	4 (3.8)
	rpsL Lys43Arg, *rrs* 888g>a	4 (3.8)
	rpsL Lys88Arg	4 (3.8)
	rpsL Lys88Arg, *rrs* 799c>t, *rrs* 888g>a	4 (3.8)
	rpsL Lys88Arg, *rrs* 888g>a	3 (2.9)
	rrs 514a>c	3 (2.9)
	rpsL Lys88Met	2 (1.9)
	gid 102del, *rpsL* Lys88Arg, *rrs* 799c>t, *rrs* 888g>a	1 (1.0)
	gid 115del	1 (1.0)
	gid 326del	1 (1.0)
	gid 351del	1 (1.0)
	rpsL Lys88Arg, *rrs* 799c>t	1 (1.0)
	rrs 462c>t, *rrs* 888g>a	1 (1.0)
	rrs 514a>c, *rrs* 799c>t	1 (1.0)
	rrs 514a>c, *rrs* 799c>t, *rrs* 888g>a	1 (1.0)
	rrs 517c>t, *rrs* 799c>t, *rrs* 888g>a	1 (1.0)
Fluoroquinolones	*gyrA* Asp94Gly	17 (43.6)
	gyrA Ala90Val	6 (15.4)

	gyrA Asp94Asn	4 (10.3)
	gyrA Ala90Val, *gyrA* Asp94Gly	2 (5.1)
	gyrA Asp94Ala	2 (5.1)
	gyrA Ala90Val, *gyrA* Asp94Ala, *gyrA* Ser91Pro	1 (2.6)
	gyrA Ala90Val, *gyrB* Asp461Asn	1 (2.6)
	gyrA Asp89Asn	1 (2.6)
	gyrA Asp94Ala, *gyrB* Asp461Ala	1 (2.6)
	gyrA Asp94Tyr	1 (2.6)
	gyrB Ala504Thr	1 (2.6)
	gyrB Glu501Asp	1 (2.6)
	gyrB Ser447Phe	1 (2.6)
Amikacin	*rrs* 1402c>a, *rrs* 1484g>t	47 (74.6)
	rrs 1402c>a	9 (14.3)
	rrs 1401a>g	4 (6.3)
	rrs 1401a>g, *rrs* 1402c>a, *rrs* 1484g>t	2 (3.2)
	rrs 1401a>g, *rrs* 1402c>a	1 (1.6)
Kanamycin	*rrs* 1402c>a, *rrs* 1484g>t	47 (72.3)
	rrs 1402c>a	9 (13.8)
	rrs 1401a>g	4 (6.2)
	rrs 1401a>g, *rrs* 1402c>a, *rrs* 1484g>t	2 (3.1)
	eis_c.-10G>A	1 (1.5)
	eis_c.-10G>A, eis_c.-14C>T	1 (1.5)
	rrs 1401a>g, *rrs* 1402c>a	1 (1.5)
Ethionamide	*fabG1* -15C>T	7 (28.0)
	ethA 1290del	1 (4.0)
	ethA 1299_1300insG	1 (4.0)
	ethA 1341del	1 (4.0)
	ethA 140del	1 (4.0)
	ethA 223del	1 (4.0)
	ethA 22del, *fabG1* -15C>T	1 (4.0)
	ethA 240del	1 (4.0)
	ethA 284_285insATCGA	1 (4.0)
	ethA 491del	1 (4.0)
	ethA 504del	1 (4.0)
	ethA 552_553insC	1 (4.0)
	ethA 620_621insG	1 (4.0)
	ethA 679del	1 (4.0)
	ethA 752_753insG	1 (4.0)
	ethA 825del	1 (4.0)
	ethA Chromosome:g.4326679_4327538del	1 (4.0)
	ethA Chromosome:g.4327134_4327434del	1 (4.0)
	fabG1 -15C>T, *inhA* Ile21Thr	1 (4.0)
Para-aminosalicylic acid	*thyX* -16C>T	3 (60.0)
	thyA His75Asn	2 (40.0)
